# A Comprehensive Molecular and Evolutionary Characterization of Chemosensory-Related Genes in *Calliptamus barbarus*

**DOI:** 10.3390/ani16142251

**Published:** 2026-07-21

**Authors:** Ye Xu, Yuzhou Zhao, Anru Lou, Rong Ji

**Affiliations:** 1 International Research Center of Cross-Border Pest Management in Central Asia, Xinjiang Key Laboratory of Special Species Conservation and Regulatory Biology, College of Life Sciences, Xinjiang Normal University, Urumqi 830017, China; xuyezdk@163.com (Y.X.); zyzdyx520@163.com (Y.Z.); 2 Tacheng, Research Field (Migratory Biology), Observation and Research Station of Xinjiang, Tacheng 834700, China; 3 College of Life Sciences, Xinjiang Normal University, Urumqi 830017, China; 4 Changji University, Changji 831100, China

**Keywords:** *Calliptamus barbarus*, chemosensory genes, evolutionary adaptation

## Abstract

*Calliptamus barbarus* (Costa, 1836) is a dominant grasshopper species in Xinjiang, characterized by high fecundity, polyphagy, and strong dispersal abilities. To investigate the molecular basis of its environmental sensing, we identified its chemosensory gene repertoire and performed a comparative analysis across six orthopteran species. We obtained 422 sequences across six major families: odorant receptors (ORs), ionotropic receptors (IRs), gustatory receptors (GRs), odorant-binding proteins (OBPs), chemosensory proteins (CSPs), and sensory neuron membrane proteins (SNMPs). Analysis of gene family dynamics identified 74 chemosensory genes associated with significant expansion events, including 40 ORs, 14 IRs, and 20 CSPs. Functional enrichment analysis revealed that these expanded gene families are associated with chemosensory behavior and olfactory transduction. Notably, core functional components such as Orco, SNMP1, and certain antennal IRs remain highly conserved across the six species. These variations in gene family size may represent molecular adaptations for environmental recognition. Our findings identify potential targets for future behavioral interference and sustainable pest management.

## Simple Summary

*Calliptamus barbarous* is a major grasshopper pest in the grasslands of Xinjiang, China, posing a substantial threat to local agriculture and livestock. The survival of this insect depends on its ability to detect chemical signals from food and mates. In this study, we identified 422 genes involved in this sensing process. By comparing *C. barbarous* with five other orthopteran species, we found that genes responsible for smelling have increased in number, while genes used for tasting have decreased to only nine. Despite these differences, the most essential genes for basic sensing remain very similar to those found in other related insects. Our results suggest that this grasshopper has evolved a specialized sense of smell to help it locate resources. These findings provide molecular insights for developing sustainable ways to manage pest outbreaks.

## 1. Introduction

*Calliptamus barbarus* (Costa, 1836) is a dominant grasshopper species in the grasslands of Xinjiang, China [1]. Its high fecundity, polyphagous feeding habits, and strong dispersal abilities allow it to maintain high population densities across the heterogeneous landscapes of both northern and southern Xinjiang [2]. These biological traits make *C. barbarus* a major threat to regional agriculture and livestock production [3]. Historically, locust management in Xinjiang has relied heavily on the intensive application of chemical pesticides, a practice that has spurred the development of insecticide resistance and compromised the integrity of the grassland ecosystem [4]. Consequently, there is an urgent need for sustainable green control strategies. Insect behavioral modulators represent a globally recognized frontier in eco-friendly pest management because they offer high sensitivity and target specificity with minimal environmental impact [5]. Deciphering the molecular mechanisms underlying chemoreception in *C. barbarus* is a critical prerequisite for identifying olfactory targets and developing novel behavioral interference technologies.

The insect chemosensory system serves as the primary interface for navigating complex environments, enabling vital activities such as host localization, mate-seeking, and predator avoidance [6]. This sensory machinery relies on the orchestrated action of several specialized protein families, including odorant-binding proteins (OBPs), chemosensory proteins (CSPs), odorant receptors (ORs), gustatory receptors (GRs), ionotropic receptors (IRs), and sensory neuron membrane proteins (SNMPs) [7,8]. These families exhibit distinct functional specialization, where OBPs and CSPs facilitate the capture and transport of hydrophobic odorants, while ORs, IRs, and GRs govern the detection of volatile chemicals and non-volatile gustatory cues. Additionally, SNMPs play an essential role in the recognition of pheromones [9,10,11,12]. The evolutionary refinement of these gene families provides the molecular foundation that allows insects to adapt to diverse ecological niches.

Recent genomic and transcriptomic breakthroughs have provided profound mechanistic insights into the nature of insect chemoreception. Structural analyses have elucidated the ion channel gating mechanisms of the *Orco* heterotetramer [13], confirming its indispensable role in pheromone detection and host selection [14,15]. Furthermore, functional studies have demonstrated that gene duplications can simultaneously enhance host localization and mate recognition [16]. The co-expression of ORs and IRs has also been shown to increase olfactory sensitivity [17], while specific IRs mediate critical avoidance behaviors [18]. Beyond sensory perception, CSPs and OBPs are increasingly implicated in physiological resilience. Certain CSPs contribute to insecticide resistance by sequestering toxic molecules and facilitating metabolic detoxification [19,20,21]. In the gustatory realm, the structural characterization of GRs has clarified how insects perceive dietary sugars and bitter compounds [22,23].

In locusts, chemosensory genes are central to the regulation of foraging, mating, and phase polyphenism. Extensive repertoires of OBP and CSP genes have been identified across various species, including *Locusta migratoria* (Linnaeus, 1758) and *Schistocerca gregaria* (Forskål, 1775) [24,25,26,27,28,29,30]. During phase transition, CSPs and *TO1* genes modulate the shift between conspecific attraction and repulsion [31]. In *Locusta migratoria*, the expression of *LmigOBP4* correlates with population density and specifically regulates gregarious behavior [32,33]. Notably, the locust olfactory system exhibits lower redundancy compared to other insect orders [34,35,36]. The aggregation pheromone 4VA triggers swarming via the specific receptor *OR35*, and its disruption can effectively reverse the gregarious phenotype [37,38]. Furthermore, receptors such as *OR70a* and *OR5* govern complex social behaviors like cannibalism and predator avoidance [36]. Functional differentiation between *SNMP1* and *SNMP2* further underscores the sophisticated regulation occurring within olfactory sensilla [39,40].

Despite these global advancements, the chemosensory system of *C. barbarus* remains poorly characterized. This study utilizes bioinformatic mining to identify six major chemosensory gene families in *C. barbarus* and subjects them to a comparative phylogenomic analysis within the context of five other orthopteran species. By integrating divergence time estimation (MCMCTree) and gene family evolution modeling, our research aims to: (1) determine the composition and diversity of the chemosensory repertoire in *C. barbarus* and (2) characterize the evolutionary dynamics and functional enrichment of these gene families within a phylogenomic framework. By establishing this molecular framework, we provide a foundation for understanding the environmental adaptation of this species and identifying potential targets for eco-friendly management.

## 2. Materials and Methods

Adult specimens of *C. barbarus* were collected in September 2024 from a desert and semi-desert steppe habitat in Urumqi, Xinjiang, China (43.53° N, 87.95° E). Individuals exhibiting vigorous locomotor activity were selected for the study. To ensure sufficient RNA yield and representative gene expression, antennae were dissected and pooled from 30–40 adult individuals (including both males and females) as a single biological sample. For the thorax, tissues were collected and combined from three adult individuals into a single sample. All tissue separation procedures were performed on ice to prevent RNA degradation. The isolated tissues were immediately stored at −80 °C for subsequent analysis.

Total RNA was extracted from the newly collected pooled antennae and thorax samples separately using TRIzol reagent (Tiangen, Beijing, China) following the manufacturer’s instructions. Independent cDNA libraries were constructed for these two tissue pools using the Clontech SMARTer PCR cDNA Synthesis Kit (Takara Bio USA, Inc., Mountain View, CA, USA). Sequencing was performed on the Illumina NovaSeq 6000 platform (San Diego, CA, USA) in 150 bp paired-end (PE150) mode. Additionally, the whole-body transcriptomic data of *C. barbarus* (accession number: SRR37687004), which were previously generated as part of our related research, were integrated into this study to ensure maximum coverage of the chemosensory gene repertoire. Following quality control and pooling of these datasets, a total of 171.33 million filtered reads (approximately 25.50 Gb of clean data) were obtained and used for subsequent de novo assembly and gene identification.

To obtain a comprehensive repertoire of chemosensory genes in *C. barbarus*, the raw reads from the newly sequenced tissues and the SRA dataset were pooled, filtered for quality, and de novo assembled using Trinity version 2.15.2 [41] with default parameters, yielding 527,600 transcripts with an N50 value of 2210 bp. To minimize sequence redundancy, these transcripts were clustered using CD-HIT-est version 4.8.1 [42] at a 95% similarity threshold, and the longest sequence from each cluster was selected as the representative unigene. Subsequently, open reading frames (ORFs) were predicted using TransDecoder version 5.7.1, with coding potential determined by the “longest ORF” rule. Finally, the completeness and integrity of the assembled transcriptome were evaluated using BUSCO version 5.7.1 [43] against the insecta_odb10 database. The assessment demonstrated a high level of transcriptome completeness, with 97.3% of the 1367 expected insect orthologs identified as complete.

To investigate the evolutionary dynamics of *C. barbarus*, we performed a comparative phylogenomic analysis using six orthopteran species (*Locusta migratoria*; *Schistocerca gregaria*; *Gryllus bimaculatus* De Geer, 1773; *Eucriotettix oculatus* Bolívar, 1898; *Laupala kohalensis* Otte, 1994; and *C. barbarus*). Detailed information regarding the data sources and assembly quality (BUSCO scores) for these six species is provided in Supplementary Table S1. Orthologous groups (OGs) were identified using OrthoFinder version 2.5.5 [44] with default parameters. A total of 308 single-copy orthologous genes were utilized for species tree reconstruction via IQ-TREE version 2.3.6 [45]. The divergence time was estimated using MCMCTree (clock = 3, RootAge ≤ 2.92, rgene gamma = 1 13.20498, sigma2 gamma = 1 4.5) from PAML version 4.10.0 [46] with the approximate likelihood method (usedata = 2). Two calibration times based on previous studies [47,48] from the TIMETREE database (www.timetree.org) were utilized for estimation: *G. bimaculatus*–*Locusta migratoria* (median: 292 Mya) and *Locusta migratoria*–*E. oculatus* (115.0–224.4 Mya).

Gene family expansion and contraction were analyzed using CAFE version 5.0 [49] based on the time-calibrated tree. Phylogenetic tree topology and branch lengths were considered when inferring the significance of changes to gene-family size in each branch. Families with conditional *p*-values lower than 0.05 were considered to have had a significantly accelerated rate of expansion or contraction. GO and KEGG enrichment analyses were performed for these significantly changed gene families using TBtools-II version 2.446 [50], and the results were visualized using the R package GOplot version 1.0.2 [51].

To identify the chemosensory gene family in *C. barbarus*, a dual-verification strategy based on sequence homology and structural conservation was implemented. First, protein sequences of known chemosensory genes from the representative orthopteran *Locusta migratoria* and *Schistocerca gregaria* were retrieved from the NCBI database and used as queries for BLAST v2.12.0 [52] searches against the *C. barbarus* protein with an E-value threshold of 10^−5^. Simultaneously, HMMER v3.0 [53] was employed to identify candidate sequences containing family-specific conserved domains by searching against the Pfam database. Finally, the intersection of the results from both BLAST and HMMER analyses was defined as the final set of candidate genes, ensuring high-confidence identification for subsequent analysis.

To investigate the phylogenetic relationships of the identified chemosensory gene subfamilies, protein sequences from the aforementioned five orthopteran species (*Locusta migratoria*, *S. gregaria*, *G. bimaculatus*, *E. oculatus*, and *Laupala kohalensis*) were used as references. Multiple sequence alignments (MSAs) of the candidate protein sequences were performed using MAFFT v7.525 [54]. The alignments were subsequently trimmed with trimAl v1.5.rev0 [55]. Maximum-likelihood (ML) phylogenetic trees were then constructed using IQ-TREE v2.3.6 [45]. The optimal substitution model for each gene family was automatically selected by ModelFinder based on the Bayesian Information Criterion (BIC). The specific models used were: JTT+G4 for ORs, Q.pfam+G4 for IRs, WAG+I+G4 for OBPs, Q.pfam+I+G4 for CSPs, Q.pfam+I+G4 for GRs, and Q.pfam+I+G4 for SNMPs. The optimal substitution model was automatically selected using the -m TEST option. Branch support was assessed with 1000 ultrafast bootstrap replicates (-bb 1000) and the SH-like approximate likelihood ratio test (-alrt 1000). The final trees were visualized via the iTOL online platform (https://itol.embl.de/, accessed on 18 July 2026).

## 3. Results and Discussion

### 3.1. Phylogenetic Relationships and Functional Enrichment of Gene Families

**Figure 1:**
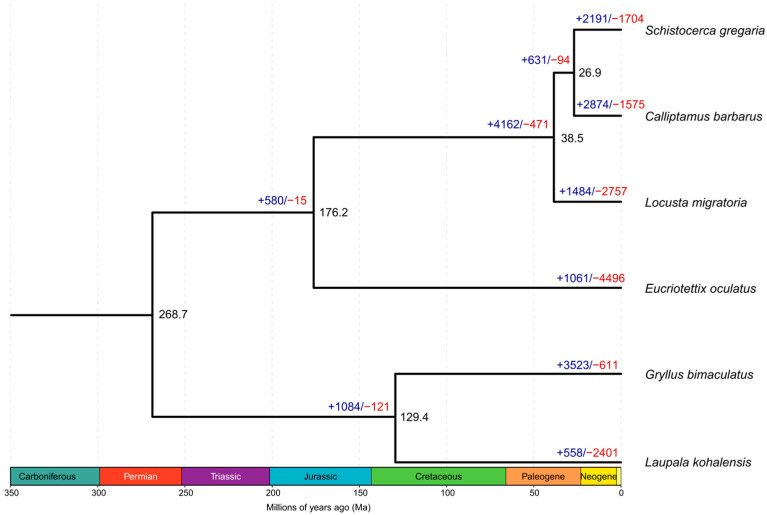
Phylogenetic tree and divergence times of the six orthopteran species. The chronogram was reconstructed using single-copy orthologs. The numbers at the nodes represent the estimated divergence times (Ma, millions of years ago) inferred by MCMCTree. Blue and red numbers on the branches indicate the counts of significantly expanded and contracted gene families (*p* < 0.05). The geological time scale at the bottom illustrates the evolutionary timeframe from the Permian to the Neogene.

A time-calibrated phylogenetic tree was reconstructed using single-copy orthologs from six orthopteran species (Figure 1). The results indicated that the suborder Ensifera (represented by *G. bimaculatus* and *Laupala kohalensis*) diverged from Caelifera by approximately 268.7 Ma. This estimate is highly consistent with our calibration constraint (median 292 Ma) and falls within the previously reported divergence range for these suborders [47,48]. Within the Caelifera, the divergence between *Locusta migratoria* and the *C. barbarus*/*S. gregaria* clade was estimated to be 38.5 Ma, while *C. barbarus* diverged from *S. gregaria* by approximately 26.9 Ma. These divergence estimates align with the rapid radiation of Acrididae during the Cenozoic and provide a temporal framework for the subsequent gene family analysis. Based on the analysis of gene family dynamics, the *C. barbarus* lineage exhibited 2874 expanded and 1575 contracted gene families (*p* < 0.05) (Figure 1).

**Figure 2:**
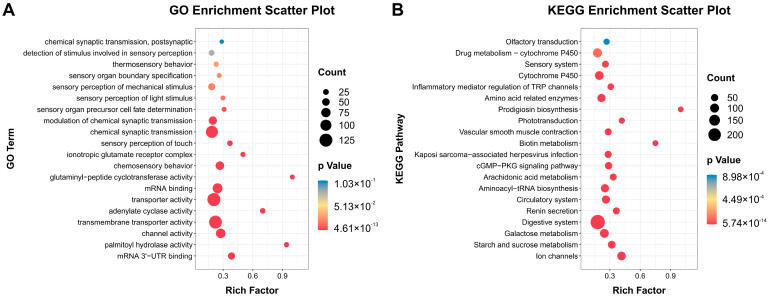
GO and KEGG enrichment of expanded gene families in *Calliptamus barbarus*. (**A**) Scatter plot of enriched GO terms in the Biological Process category. (**B**) Scatter plot of enriched KEGG pathways. In both panels, the *x*-axis represents the Rich Factor, which indicates the degree of enrichment. The size of the bubbles corresponds to the number of expanded genes associated with each term, while the color gradient represents the *p*-value.

To characterize the functional distribution of these expanded families, GO and KEGG enrichment analyses were performed. In the GO analysis, expanded gene families were associated with several sensory-related terms, including chemosensory behavior (GO:0007635, *p* = 3.17 × 10^−8^), sensory perception of touch (GO:0050975, *p* = 1.82 × 10^−5^), and ionotropic glutamate receptor complex (GO:0008328, *p* = 1.69 × 10^−6^) (Figure 2A, Table S3). KEGG pathway analysis revealed enrichment in ion channels (ko04040, *p* = 5.74 × 10^−14^), phototransduction (ko04744, *p* = 2.90 × 10^−7^), cytochrome P450 (ko00199, *p* = 6.33 × 10^−6^), and olfactory transduction (ko04740, *p* = 8.98 × 10^−4^) (Figure 2B, Table S4). Other enriched categories included thermosensory behavior (GO:0040040) and inflammatory mediator regulation of TRP channels (ko04750) (Figure 2B, Table S4). In the GO analysis, contracted gene families were enriched in categories related to enzymatic activities and cellular components, such as protein kinase activity (GO:0004672, *p* = 0.00097) and structural constituent of muscle (GO:0008307, *p* = 1.58 × 10^−9^) (Supplementary Figure S1A, Table S5). Similarly, KEGG pathway analysis identified several pathways associated with basic physiological and metabolic processes, including galactose metabolism (ko00052, *p* = 1.71 × 10^−14^) and carbohydrate digestion and absorption (ko04973, *p* = 2.57 × 10^−14^) (Supplementary Figure S1B, Table S6).

### 3.2. Comparative Analysis of Chemosensory Gene Families

In this study, six major chemosensory gene families were identified in *C. barbarus*: ORs, IRs, GRs, OBPs, CSPs, and SNMPs. We characterized the repertoire sizes of these families through a comparative analysis with five other orthopteran species across three families: Acrididae, Tetrigidae, and Gryllidae (Table 1). The OR family in *C. barbarus* consists of 287 candidate genes, which is higher than the counts for *Locusta migratoria* (146) and *S. gregaria* (208) and exceeds the numbers identified in the crickets *G. bimaculatus* (31) and *Laupala kohalensis* (19). In contrast, the SNMP family remains relatively uniform across the six species, with *C. barbarus* possessing 16 members, a count comparable to those for *Locusta migratoria* (13) and *Laupala kohalensis* (18). Within other receptor families, IRs and GRs show different patterns. The IR repertoire in *C. barbarus* (55 genes) is similar to those of *S. gregaria* (59) and *E. oculatus* (63) but higher than in *Locusta migratoria* (30). Conversely, the GR repertoire in *C. barbarus* is limited to nine genes, which is fewer than in *Locusta migratoria* (16) and lower than in the tetrigid *E. oculatus* (52). Regarding soluble carrier proteins, the *C. barbarus* OBP family includes 32 members, the highest count among the six species. Meanwhile, the CSP family in *C. barbarus* (23 genes) is larger than in *Locusta migratoria* (15) but smaller than in *S. gregaria* (39) (Table 1). In summary, the chemosensory gene families in *C. barbarus* exhibit variations in repertoire size that are broadly consistent with the lineage-specific expansions and contractions identified in our phylogenomic analysis. To further resolve these evolutionary trends, the intersection between the globally expanded/contracted gene sets and the chemosensory repertoire was visualized using Venn diagrams (Supplementary Figures S2 and S3). Through this cross-referencing, we identified 74 expanded chemosensory genes (40 ORs, 14 IRs, and 20 CSPs) and four contracted genes (one GR and three SNMPs). The list of these genes, including their orthogroup IDs and *p*-values, is provided in Supplementary Table S2.

### 3.3. Phylogenetic Analysis of Candidate Odorant Receptors (ORs)

**Figure 3:**
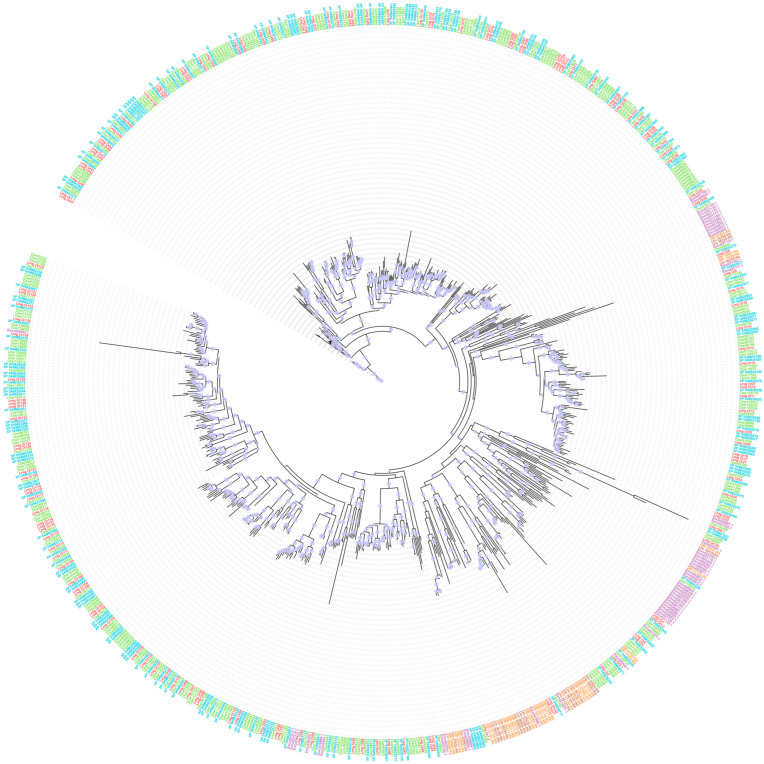
Phylogenetic tree of putative odorant receptors (ORs). Maximum-likelihood tree of putative ORs from *Calliptamus barbarus* and other representative orthopteran species. Branch support was estimated using 1000 bootstrap replicates. Colors indicate species origin as follows: *C. barbarus* (green), *Locusta migratoria* (red), *Schistocerca gregaria* (cyan), *Gryllus bimaculatus* (gold), *Laupala kohalensis* (brown), and *Eucriotettix oculatus* (purple).

We performed a phylogenetic analysis of the 287 candidate ORs identified in *C. barbarus*. A maximum-likelihood (ML) tree was constructed using these sequences along with the OR repertoires from five other orthopteran species (*Locusta migratoria*, *S. gregaria*, *G. bimaculatus*, *E. oculatus*, and *Laupala kohalensis*) (Figure 3). The phylogenetic tree showed a highly supported *Orco* clade (bootstrap = 100). Within this group, *Cbar7781* clustered with orthologs from the other five species, including *Lmig_Orco*, *Eocu014201.1*, and *GBI_14130-RA*. Larsson et al. previously confirmed that *Orco* is highly conserved across diverse insect orders and serves as the core component of functional receptor complexes [56]. Our results indicate that the fundamental olfactory transduction mechanism remains stable in *C. barbarus*, and this conservation confirms the reliability of our phylogenetic reconstruction.

Compared to the other surveyed species, the *C. barbarus* OR repertoire exhibits substantial variation in size. The 287 candidate ORs identified in this study are more numerous than those in *Locusta migratoria* (146) and *S. gregaria* (208) and notably exceed the counts in *E. oculatus* (57), *G. bimaculatus* (31), and *Laupala kohalensis* (19) (Table 1). Among these candidate ORs in *C. barbarus*, 40 genes belong to significantly expanded orthogroups (*p* < 0.05; Supplementary Table S2). This diversity in repertoire size follows the “birth-and-death” model of chemosensory gene evolution, where gene duplication and loss events shape species-specific receptor sets [57].

*Calliptamus barbarus* is a dominant species in the arid and semi-arid grasslands of Xinjiang, characterized by a wide distribution of and exposure to complex vegetation. Robertson noted that the scale of receptor family expansion generally correlates with the width of a species’ chemosensory niche [8]. Therefore, such an extensive OR repertoire may provide the molecular basis for detecting diverse host plants and their secondary metabolites, which is consistent with the polyphagous feeding habits of this species.

The 40 expanded OR genes are predominantly organized into dense, lineage-specific clusters in the ML tree (e.g., clades containing *Cbar72990*–*Cbar72999* and *Cbar89617*–*Cbar89629*), suggesting relatively recent and rapid divergence. Olfactory evolution is often driven by a species’ specific ecological requirements [58]. For *C. barbarus*, which exhibits strong dispersal capabilities and high reproductive rates, efficient long-range olfaction potentially aids in locating suitable host patches within vast habitats. Furthermore, these divergent OR clades may be involved in mate recognition and oviposition site selection. This functional differentiation potentially supports the species’ ability to maintain population dominance in the patchy and fluctuating environments of Xinjiang. In summary, the expansion and divergence of the *C. barbarus* OR family are consistent with its polyphagy and dispersal capacity, representing a possible evolutionary adaptation to complex and heterogeneous arid environments.

### 3.4. Phylogenetic Analysis of Candidate Ionotropic Receptors (IRs)

**Figure 4:**
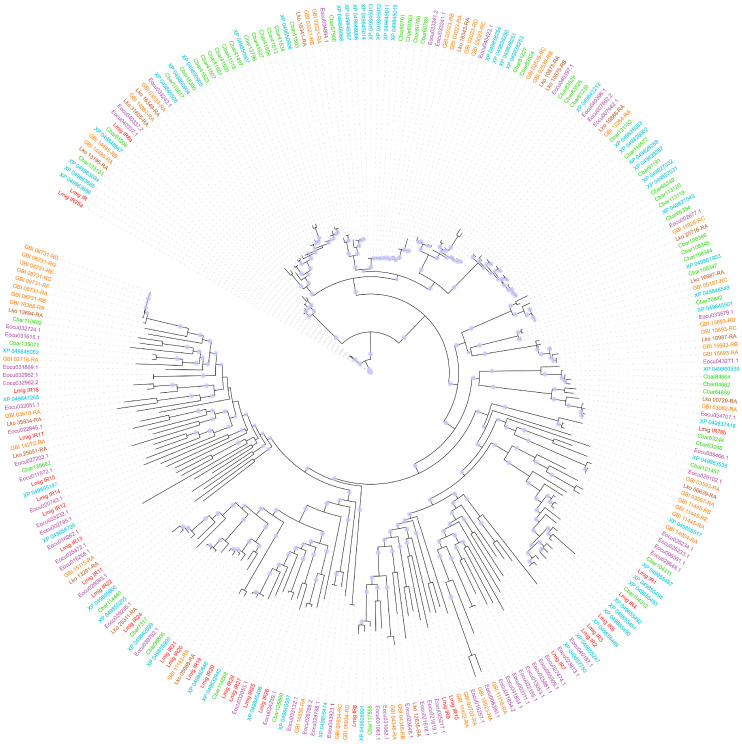
Phylogenetic tree of putative ionotropic receptors (IRs). Maximum-likelihood tree of putative IRs from *Calliptamus barbarus* and other representative orthopteran species. Branch support was estimated using 1000 bootstrap replicates. Colors indicate species origin as follows: *C. barbarus* (green), *Locusta migratoria* (red)*, Schistocerca gregaria* (cyan), *Gryllus bimaculatus* (gold), *Laupala kohalensis* (brown), and *Eucriotettix oculatus* (purple).

We analyzed the evolutionary relationships of the 55 candidate IRs identified in *C. barbarus* by constructing a maximum likelihood (ML) tree alongside repertoires from six orthopteran species (Figure 4). As established in previous studies, chemosensory gene repertoires are shaped by the interplay between evolutionary “chance” and biological “necessity” [59]. Phylogenetic analysis identified three highly supported clades corresponding to the conserved insect IR co-receptors: *Cbar133101* (IR25a), *Cbar61908* (IR8a), and *Cbar63246* (IR76b). This finding aligns with recent genome-wide identifications in other insects, which highlight the functional requirement of these co-receptors in forming multimeric sensing complexes [60]. These core components represent stable dimensions in chemosensory genetics, maintaining high sequence conservation to safeguard fundamental sensory integrity [61].

Notably, a subset of *C. barbarus* IRs clustered within the “antennal IR” subgroup, exhibiting clear orthologous relationships with receptors from *Locusta migratoria* and *S. gregaria* (e.g., *Cbar7317* clustered with *Lmig_IR24* and *Eocu039762.1*). These conserved lineages typically perform stable olfactory functions across different orthopteran families. The conservation of this repertoire suggests that *C. barbarus* retains essential sensory pathways for detecting common environmental chemical cues.

In contrast to the conserved core, a substantial portion of the *C. barbarus* IR repertoire belongs to the “divergent IR” group. Specifically, 14 IR genes were found to belong to orthogroups that underwent significant expansion (*p* < 0.05; Supplementary Table S2). This expansion is prominent in several lineage-specific clusters, such as the *Cbar41497–Cbar41534* cluster (Figure 4). This group shows prominent lineage-specific expansion within the Acrididae branch. Such chemosensory restructuring is often a hallmark of local adaptation [62], as these divergent sets evolve dynamically through frequent gene gain and loss [63]. Recent genomic analyses of the orthopteroid pest *Gryllotalpa orientalis* also indicate that niche-specific expansions provide the sensory flexibility necessary for specialized habitats [64]. For *C. barbarus*, the expansion of these divergent IR genes potentially facilitates the navigation of the complex chemical ecology in arid grassland ecosystems.

### 3.5. Phylogenetic Analysis of Candidate Odorant-Binding Proteins (OBPs)

**Figure 5:**
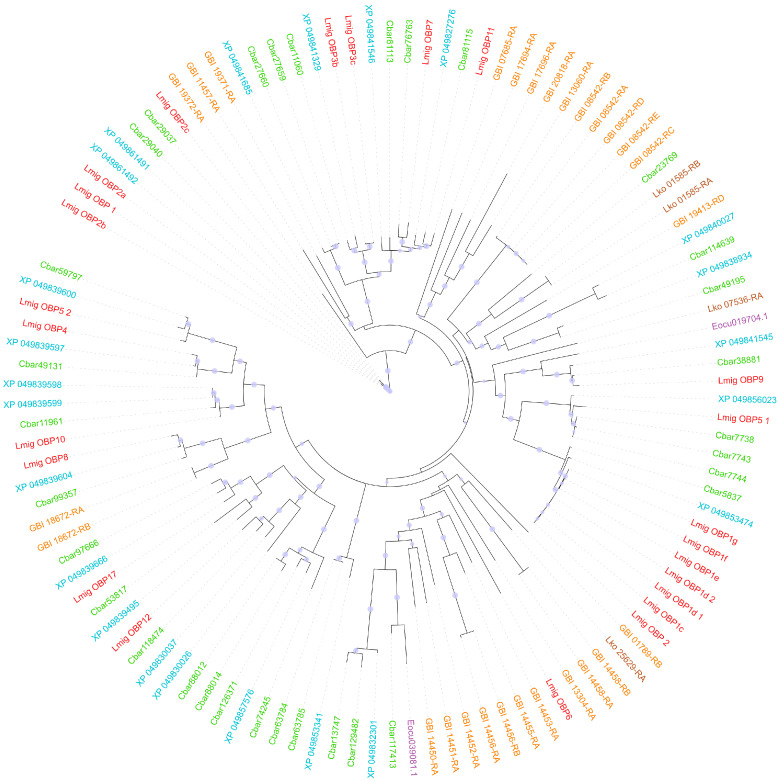
Phylogenetic tree of putative odorant-binding proteins (OBPs). Maximum-likelihood tree of putative OBPs from *Calliptamus barbarus* and other representative orthopteran species. Branch support was estimated using 1000 bootstrap replicates. Colors indicate species origin as follows: *C. barbarus* (green), *Locusta migratoria* (red), *Schistocerca gregaria* (cyan), *Gryllus bimaculatus* (gold), *Laupala kohalensis* (brown), and *Eucriotettix oculatus* (purple).

We constructed a maximum-likelihood (ML) tree to analyze the evolutionary relationships of the 32 candidate OBPs identified in *C. barbarus* alongside repertoires from five other species (Figure 5). OBPs are small, soluble proteins that transport hydrophobic odorants from the environment to membrane-bound receptors [65,66]. Phylogenetic analysis showed that several *C. barbarus* OBPs clustered with those of *Locusta migratoria* in highly supported clades. For instance, *Cbar29037* and *Cbar29040* exhibited high homology with the *Lmig_OBP2* group, and *Cbar11060* clustered with *Lmig_OBP3b/c*. These stable orthologous relationships indicate that certain OBP lineages have been conserved within the Orthoptera, likely performing fundamental physiological roles in detecting common chemical signals.

The *C. barbarus* OBP repertoire (32 genes) is the largest among the six studied species, exceeding the counts for *Locusta migratoria* (24), *S. gregaria* (23), *G. bimaculatus* (27), *Laupala kohalensis* (4), and *E. oculatus* (2) (Table 1). Notably, the OBP family has not expanded as substantially as the OR family. This asymmetric expansion between binding proteins and receptors suggests that a relatively small set of OBPs may support a diverse receptor repertoire by utilizing broad ligand-binding spectra [67]. Within the LUSH (also known as OBP76a) clade, we identified a *C. barbarus* ortholog, *Cbar38881*, which clustered with *Lmig_OBP9*. Studies in *Drosophila* Fallén, 1823, confirmed that LUSH is required for the activation of pheromone-sensitive neurons [68]. The presence of this ortholog suggests that *C. barbarus* retains specialized mechanisms for intra-specific chemical communication.

The variation in the *C. barbarus* OBP repertoire size is consistent with its polyphagous feeding strategy. As a dominant species across the grasslands of Xinjiang, this species encounters a wide array of plant secondary metabolites. A more diverse OBP set potentially provides the biochemical flexibility necessary to effectively solubilize and transport various hydrophobic ligands. This sensory adaptation works in coordination with the expanded receptor system and might be associated with the ecological success and dispersal capabilities of the species in heterogeneous environments.

### 3.6. Phylogenetic Analysis of Candidate Chemosensory Proteins (CSPs)

**Figure 6:**
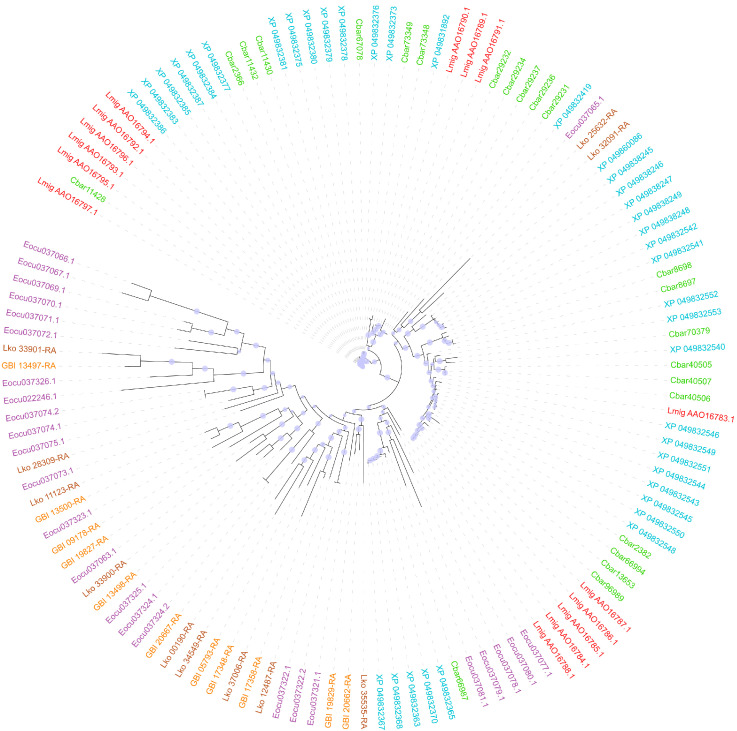
Phylogenetic tree of putative chemosensory proteins (CSPs). Maximum-likelihood tree of putative CSPs from *Calliptamus barbarus* and other representative orthopteran species. Branch support was estimated using 1000 bootstrap replicates. Colors indicate species origin as follows: *C. barbarus* (green), *Locusta migratoria* (red), *Schistocerca gregaria* (cyan), *Gryllus bimaculatus* (gold), *Laupala kohalensis* (brown), and *Eucriotettix oculatus* (purple).

We constructed a maximum-likelihood (ML) tree to analyze the evolutionary relationships of the 23 candidate CSPs identified in *C. barbarus*, comparing them with five other orthopteran species (Figure 6). CSPs, originally identified as the OS-D-like gene family, are characterized by a conserved four-cysteine motif and represent a class of highly stable soluble proteins [69]. Phylogenetic analysis showed that several *C. barbarus* CSPs formed highly supported orthologous clades with sequences from other species, such as the pair involving *Cbar2366* and *Lmig_CSP_AAO16797.1*. These conserved lineages likely represent an ancestral protein set performing fundamental physiological functions across diverse orthopteran species. The *C. barbarus* CSP repertoire (23 genes) is larger than those of *Locusta migratoria* (15), *G. bimaculatus* (11), and *Laupala kohalensis* (11), though it remains smaller than the count for *S. gregaria* (39) (Table 1). Notably, 20 out of the 23 identified CSP genes (87%) were associated with significant expansion signals (*p* < 0.05; Supplementary Table S2). In the ML tree, this expansion is manifested in several dense, species-specific clusters, most notably the *Cbar29231–Cbar29237*, *Cbar40505–Cbar40507*, and *Cbar11428–Cbar11432* clades (Figure 6). While OBP and CSP families in other insects often exhibit adaptive patterns linked to their ecological niches [70], the nearly family-wide expansion in *C. barbarus* is striking. Previous studies have indicated that CSPs are frequently involved in host plant recognition and environmental sensing [71]. The expansion of these specific CSP lineages potentially enhances the sensory adaptation of *C. barbarus*. This genomic feature may be associated with the species’ high reproductive rates and dispersal capabilities, potentially facilitating efficient host and mate localization across the diverse and heterogeneous arid habitats of Xinjiang.

### 3.7. Phylogenetic Analysis of Candidate Gustatory Receptors (GRs)

**Figure 7:**
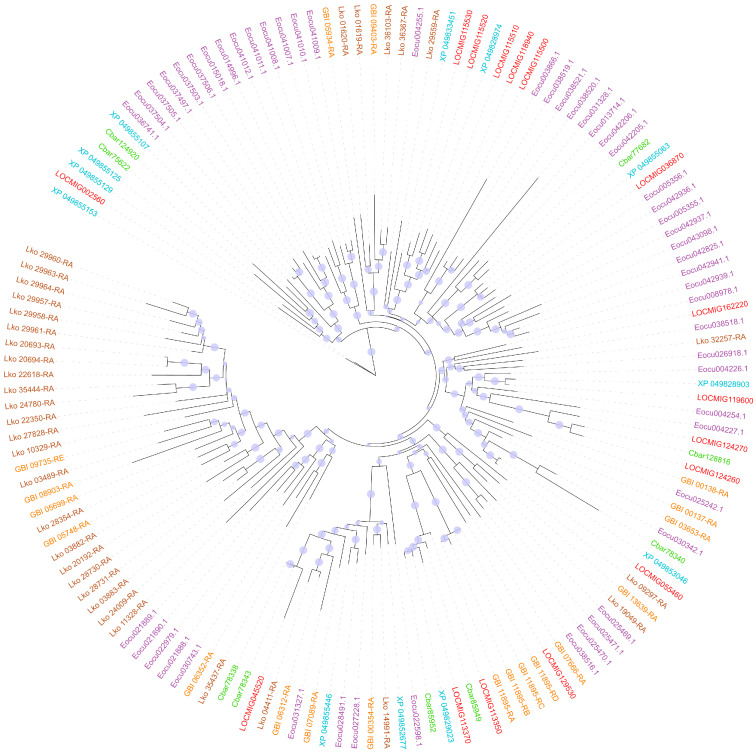
Phylogenetic tree of putative gustatory receptors (GRs). Maximum-likelihood tree of putative GRs from *Calliptamus barbarus* and other representative orthopteran species. Branch support was estimated using 1000 bootstrap replicates. Colors indicate species origin as follows: *C. barbarus* (green), *Locusta migratoria* (red), *Schistocerca gregaria* (cyan), *Gryllus bimaculatus* (gold), *Laupala kohalensis* (brown), and *Eucriotettix oculatus* (purple).

We analyzed the evolutionary relationships of the nine candidate GRs identified in *C. barbarus* by constructing a maximum-likelihood (ML) tree including repertoires from five other orthopteran species (Figure 7). Although the *C. barbarus* GR family is substantially smaller than those of *E. oculatus* (52 genes) and *Laupala kohalensis* (34 genes), these genes represent a core repertoire of functionally critical receptors [72]. Phylogenetic analysis identified several orthologs associated with fundamental sensory functions. *Cbar85949* and *Cbar85952* clustered within the conserved CO_2_ receptor clade, which mediates carbon dioxide detection in insects [10]. Within the sugar receptor group, we identified *Cbar78338* and *Cbar78343* (Figure 7), which are involved in perceiving sweet compounds [23]. Notably, *Cbar128816* was identified as an ortholog of the Gr28a/b complex, which is associated with the avoidance of aversive compounds [73]. Additionally, *Cbar77682* and *Cbar78340* were identified as orthologs within the fructose receptor lineage [11].

The overall contraction of the *C. barbarus* GR repertoire is prominent compared to the expansion of its OR and CSP families. Beyond these core nutrient and CO_2_ sensors, *C. barbarus* lacks the extensive bitter receptor radiations found in some other insect lineages [12,74]. This streamlined receptor set is consistent with a sensory strategy that prioritizes primary nutrient detection and internal homeostasis over a complex array of bitter compounds [9]. Notably, *Cbar124920* was identified within a significantly contracted orthogroup (*p* < 0.05; Supplementary Table S2), indicating a targeted reduction within this family in the *C. barbarus* lineage. The retention of essential pathways for detecting common toxins or aversive stimuli suggests that *C. barbarus* maintains a functional baseline for gustatory perception in arid and semi-arid environments.

### 3.8. Phylogenetic Analysis of Candidate Sensory Neuron Membrane Proteins (SNMPs)

**Figure 8:**
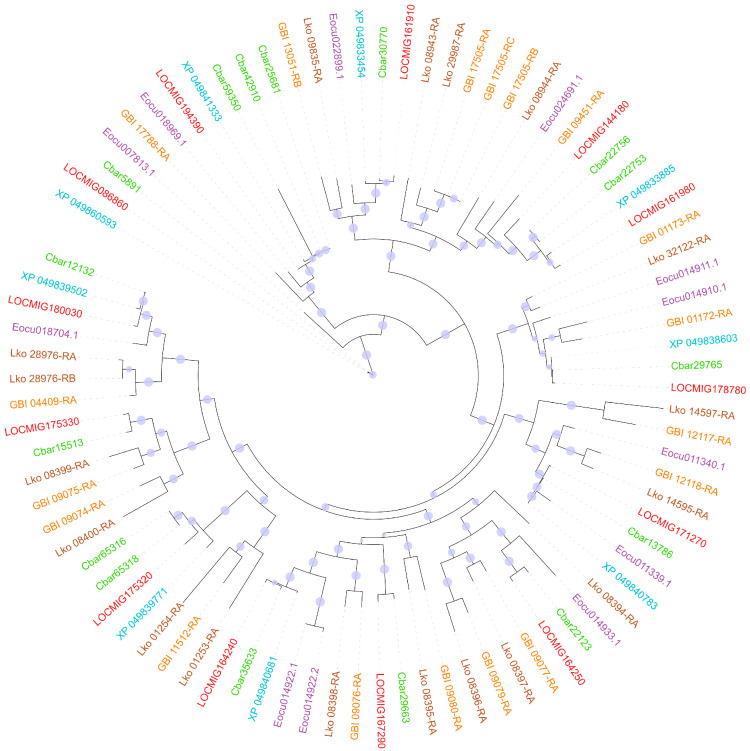
Phylogenetic tree of putative sensory neuron membrane proteins (SNMPs). Maximum-likelihood tree of putative SNMPs from *Calliptamus barbarus* and other representative orthopteran species. Branch support was estimated using 1000 bootstrap replicates. Colors indicate species origin as follows: *C. barbarus* (green), *Locusta migratoria* (red), *Schistocerca gregaria* (cyan), *Gryllus bimaculatus* (gold), *Laupala kohalensis* (brown), and *Eucriotettix oculatus* (purple).

We analyzed the evolutionary relationships of the 16 candidate SNMPs identified in *C. barbarus* by constructing a maximum-likelihood (ML) tree including repertoires from five other orthopteran species (Figure 8). SNMPs represent a specialized group within the CD36 gene superfamily [75]. Phylogenetic analysis accurately categorized the *C. barbarus* SNMPs into established subgroups. Specifically, *Cbar25681*, *Cbar42910*, and *Cbar59350* clustered within the SNMP1 clade with high bootstrap support (Figure 8). Studies in other insects have confirmed that SNMP1 is essential for detecting lipid-derived pheromones [76], and these proteins are typically localized in pheromone-sensitive neurons [77]. The remaining *C. barbarus* sequences clustered with the SNMP2 lineage or other CD36-like proteins, which are often involved in broader metabolic functions [78].

In contrast to the lineage-specific expansions observed in the OR and CSP families, the SNMP family remains relatively uniform across the six compared orthopteran species. *C. barbarus* possesses 16 SNMPs, while the counts in *Locusta migratoria* (13), *G. bimaculatus* (12), and *Laupala kohalensis* (18) are comparable (Table 1). This numerical stability suggests that SNMPs are under strong selective constraints and experience lower rates of gene gain and loss compared to other chemosensory families [79]. Within this family, three genes (*Cbar22753*, *Cbar22756*, and *Cbar30770*) were associated with significant contraction signals (*p* < 0.05; Supplementary Table S2). This indicates that while the essential SNMP repertoire is well-maintained, certain lineage-specific variants have been lost in *C. barbarus*. Overall, the conservation of core SNMP lineages suggests that *C. barbarus* retains the fundamental molecular mechanisms necessary for pheromone signaling and lipid metabolism.

### 3.9. Limitations and Future Prospects

Although this study provides a comprehensive overview of the *C. barbarus* chemosensory repertoire, several limitations remain. The use of transcriptomic data can under-represent genes with low expression levels or those restricted to specific developmental stages. Furthermore, the reliance on transcriptomes for *C. barbarus* versus genomic datasets for reference species could introduce systematic bias in estimating gene family expansion and contraction rates. To address these gaps, future research incorporating chromosome-level genome sequencing will be important for a more definitive characterization. Moreover, functional validation through techniques such as RNA interference and electrophysiology is still necessary to confirm the specific roles of these candidate genes in environmental sensing.

## 4. Conclusions

In this study, we identified a repertoire of 422 candidate chemosensory genes in *C. barbarus*. Repertoire sizes varied among gene families, with the OR and CSP families exhibiting clear lineage-specific expansions, while the GR family contained only nine members. Despite these differences, core chemosensory genes such as *Orco*, *SNMP1*, and certain antennal IRs remain highly conserved across the six studied species. This combination of a stable molecular core and a diversified peripheral gene set suggests that *C. barbarus* has retained fundamental sensory pathways while adapting its environmental recognition capabilities. These genomic traits may contribute to the ecological success of *C. barbarus* in the arid and semi-arid grassland ecosystems of Xinjiang. The expansion of specific OR and CSP lineages points to an enhanced capacity for recognizing host plant volatiles, which potentially facilitates efficient resource localization across heterogeneous landscapes. Overall, our findings provide a molecular framework for understanding the sensory evolution of *C. barbarus* and identify potential targets for future behavioral interference and sustainable pest management.

## Figures and Tables

**Table 1:** Numbers of each chemosensory-related gene subfamily in six orthopteran species.

Families	*Locusta* *migratoria*	*Calliptamus* *barbarus*	*Schistocerca gregaria*	*Gryllus* *bimaculatus*	*Eucriotettix* *oculatus*	*Laupala* *kohalensis*
OR	146	287	208	31	57	19
IR	30	55	59	48	63	20
OBP	24	32	23	27	2	4
CSP	15	23	39	11	26	11
GR	16	9	12	19	52	34
SNMP	13	16	9	18	12	18

Note: Data for *Calliptamus barbarus* were derived from transcriptome assembly. Repertoires for the other five orthopteran species (*Locusta migratoria*, *Schistocerca gregaria*, *Gryllus bimaculatus*, *Eucriotettix oculatus*, and *Laupala kohalensis*) were retrieved from genomic datasets.

## Data Availability

The raw transcriptomic data for *Calliptamus barbarus* generated in this study have been deposited in the NCBI Sequence Read Archive (SRA) under BioProject accession number PRJNA1447077. Specifically, the datasets for the thorax and antennae are available under the accession numbers SRR37899237 and SRR37899238, respectively.

## Supplementary Materials

The following supporting information can be downloaded at https://www.mdpi.com/article/10.3390/ani16142251/s1, Figure S1. GO and KEGG enrichment of contracted gene families in Calliptamus barbarus. (A) Scatter plot of enriched GO terms in the Biological Process category. (B) Scatter plot of enriched KEGG pathways. In both panels, the *x*-axis represents the Rich Factor, which indicates the degree of enrichment. The size of the bubbles corresponds to the number of contracted genes associated with each term, while the color gradient represents the *p*-value. Figure S2. Overlap between expanded gene families and chemosensory subfamilies. Figure S3. Overlap between contracted gene families and chemosensory subfamilies. Table S1. Data sources and BUSCO assessment of six orthopteran species. Table S2. Identification of expanded and contracted chemosensory genes in *Calliptamus barbarus*. Table S3. GO enrichment of expanded gene families in *Calliptamus barbarus*. Table S4. KEGG enrichment of expanded gene families in *Calliptamus barbarus*. Table S5. GO enrichment of contracted gene families in *Calliptamus barbarus*. Table S6. KEGG enrichment of contracted gene families in *Calliptamus barbarus*.
