# Supplementary material for: A Comprehensive Molecular and Evolutionary Characterization of Chemosensory-Related Genes in Calliptamus barbarus
Source: Animals (Basel). 2026 Jul 21;16(14):2251. doi: 10.3390/ani16142251 (PMC13404529; doi:10.3390/ani16142251)
Supplement: Supplementary file 1 [file animals-16-02251-s001.zip › Supplemental Information-Figure S1-S3.pdf]

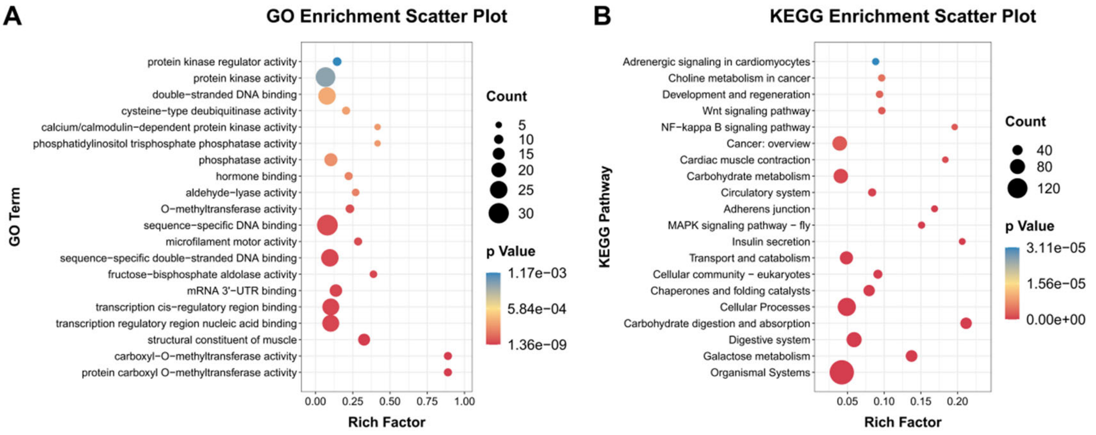

Figure S1. GO and KEGG enrichment of contracted gene families in *Calliptamus barbarus*. (A) Scatter plot of enriched GO terms in the Biological Process category. (B) Scatter plot of enriched KEGG pathways. In both panels, the x-axis represents the Rich Factor, which indicates the degree of enrichment. The size of the bubbles corresponds to the number of contracted genes associated with each term, while the color gradient represents the *p*-value.

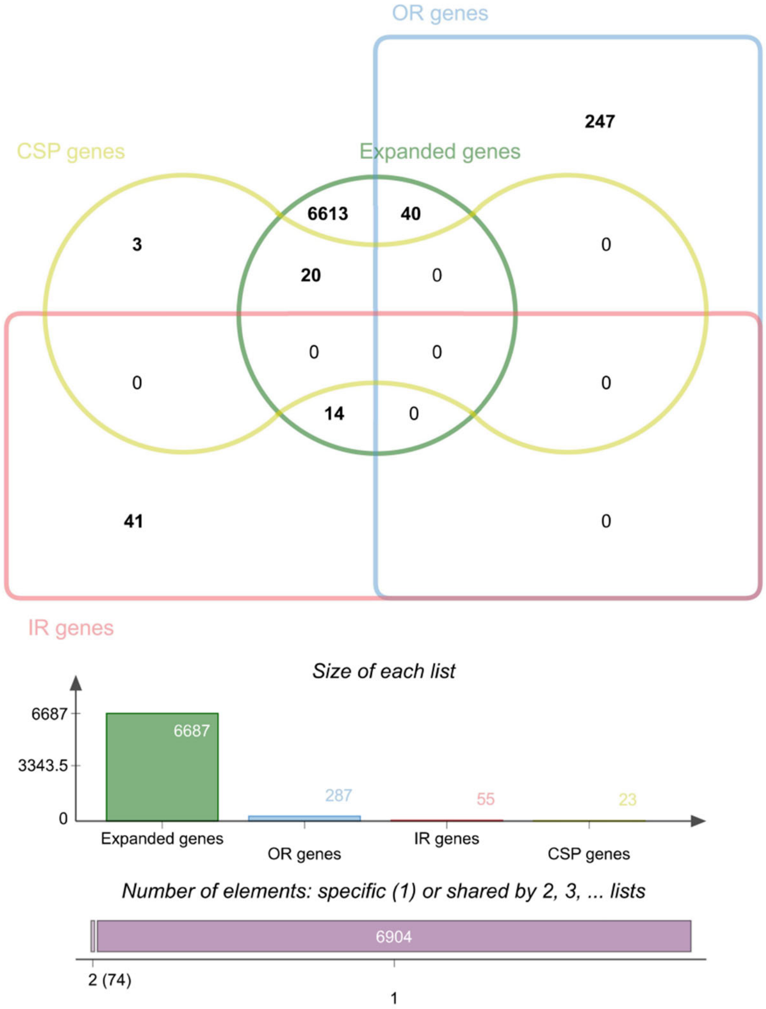

Figure S2. Overlap between expanded gene families and chemosensory subfamilies.

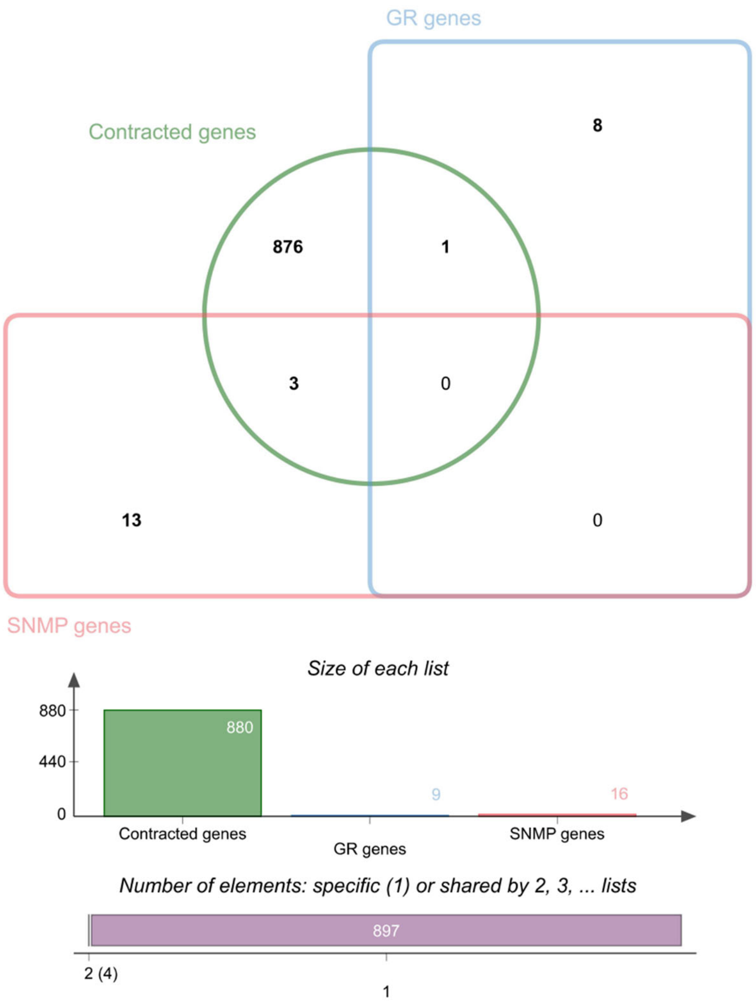

Figure S3. Overlap between contracted gene families and chemosensory subfamilies.
